# Robotic pedicle screw placement for minimal invasive thoracolumbar spine surgery: a technical note

**DOI:** 10.3389/fsurg.2024.1495251

**Published:** 2025-01-20

**Authors:** Luthfi Gatam, Phedy Phedy, Syafruddin Husin, Harmantya Mahadhipta, Asrafi Rizki Gatam, Mitchel Mitchel, Karina Sylvana Gani, Erica Kholinne

**Affiliations:** ^1^ Department of Orthopedics, Gatam Institute, Tangerang, Indonesia; ^2^ Department of Orthopedic Surgery, Fatmawati Hospital, Jakarta, Indonesia; ^3^ Department of Orthopedic Surgery, Premier Bintaro Hospital, Tangerang, Indonesia; ^4^ Department of Orthopedic Surgery, Faculty of Medicine, Universitas Trisakti, Jakarta, Indonesia

**Keywords:** thoracolumbar spine, pedicle screw placement, robotic spine surgery, ExcelsiusGPS robot, minimally invasive

## Abstract

**Background:**

Pedicle screw placement in spine surgery is a complex and delicate procedure that requires precise and accurate placement of pedicle screws. This technical note describes the steps involved in performing robotic assistance pedicle screw insertion in thoracolumbar spine surgery using the ExcelsiusGPS platform.

**Methods:**

This paper outlines the surgical techniques and intraoperative workflow for pedicle screw placement using the ExcelsiusGPS system. It also covers the surgical process, including patient positioning, dynamic reference placement, intraoperative cone-beam tomography, screw planning, exposure, and insertion techniques for spinal stabilization.

**Discussion:**

A meta-analysis highlighted the significant advantages of robotic spine surgery over traditional freehand techniques, including a notably lower complication rate (4.83% vs. 14.97%) and up to a tenfold reduction in surgeon radiation exposure compared to fluoroscopy. Additionally, robotic systems enhance pedicle screw placement accuracy, achieving a 91.7% success rate. This higher accuracy is attributed to real-time screw planning, trajectory guidance, and precise adjustments in robotic-assisted surgery. These advantages establish robotic assistance as a crucial innovation for enhancing surgical precision and patient safety, although it requires careful handling of technical challenges like alignment changes in highly flexible bones and ensuring accurate instrument trajectory during screw placement.

**Conclusion:**

Robotic-assisted spine surgery improves pedicle screw accuracy with real-time planning and trajectory adjustments, reducing complications and radiation exposure. However, higher costs and increased screw use warrant careful evaluation of its cost-effectiveness and impact on healthcare resources.

## Introduction

Spine surgery is a complex and delicate medical procedure that sometimes requires precise and accurate pedicle screw placement to ensure successful outcomes. Pedicle screw malposition can lead to severe neurovascular injuries (1–4). Efficient and accurate screw placement is fundamental in reducing iatrogenic complications and improving surgical outcomes. The limitations of human hands and visualization in achieving such precision can be challenging. Robotic assistance in spinal surgery minimizes these human errors. It ensures patient safety (5). Robot-assisted pedicle screw insertion has been introduced, to improve accuracy (6). Pedicle screw misplacement rates using conventional techniques range from 30% in the lumbar spine to 55% in the thoracic spine (7, 8). In contrast, a study has demonstrated that robotic-assisted pedicle screw placement achieves success rates exceeding 90% (9). A recent meta-analysis revealed that robotic-assisted techniques have a significantly lower complication rate compared to the freehand method (4.83% vs. 14.97%). However, the robotic-assisted group did not show a significant advantage in clinical efficacy (10). This technical note aims to describe the steps in performing robotic assistance pedicle screw placement in thoracolumbar spine surgery.

## Materials and equipments

### Robotic device and patient positioning

**Figure 1:**
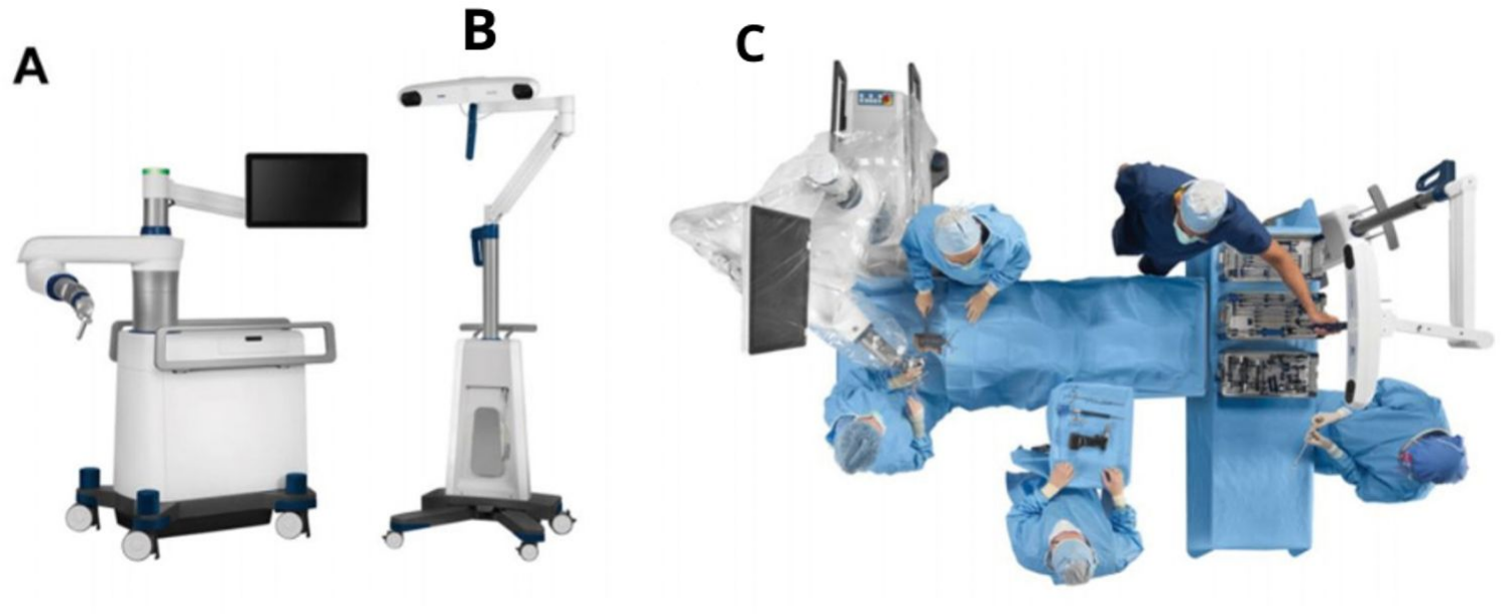
**(A)** ExclesiusGPS with a floor-fixable base and rigid robotic arm. **(B)** Navigation camera **(C)** Simulated operating theater using ExclesiusGPS (11).

**Figure 2:**
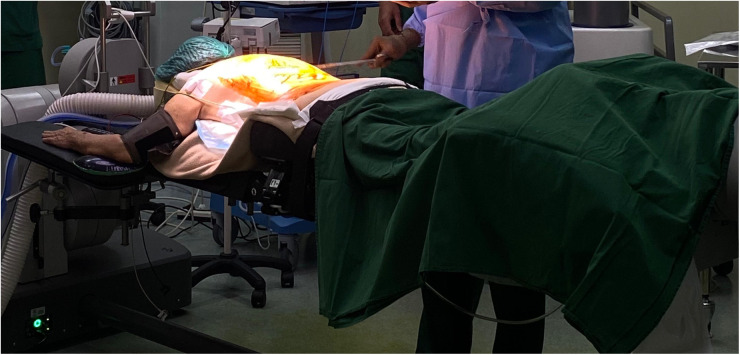
The patient is positioned on a Jackson table. A cushion and an armrest can be used under the patient to ensure proper positioning and comfort during the procedure.

**Figure 3:**
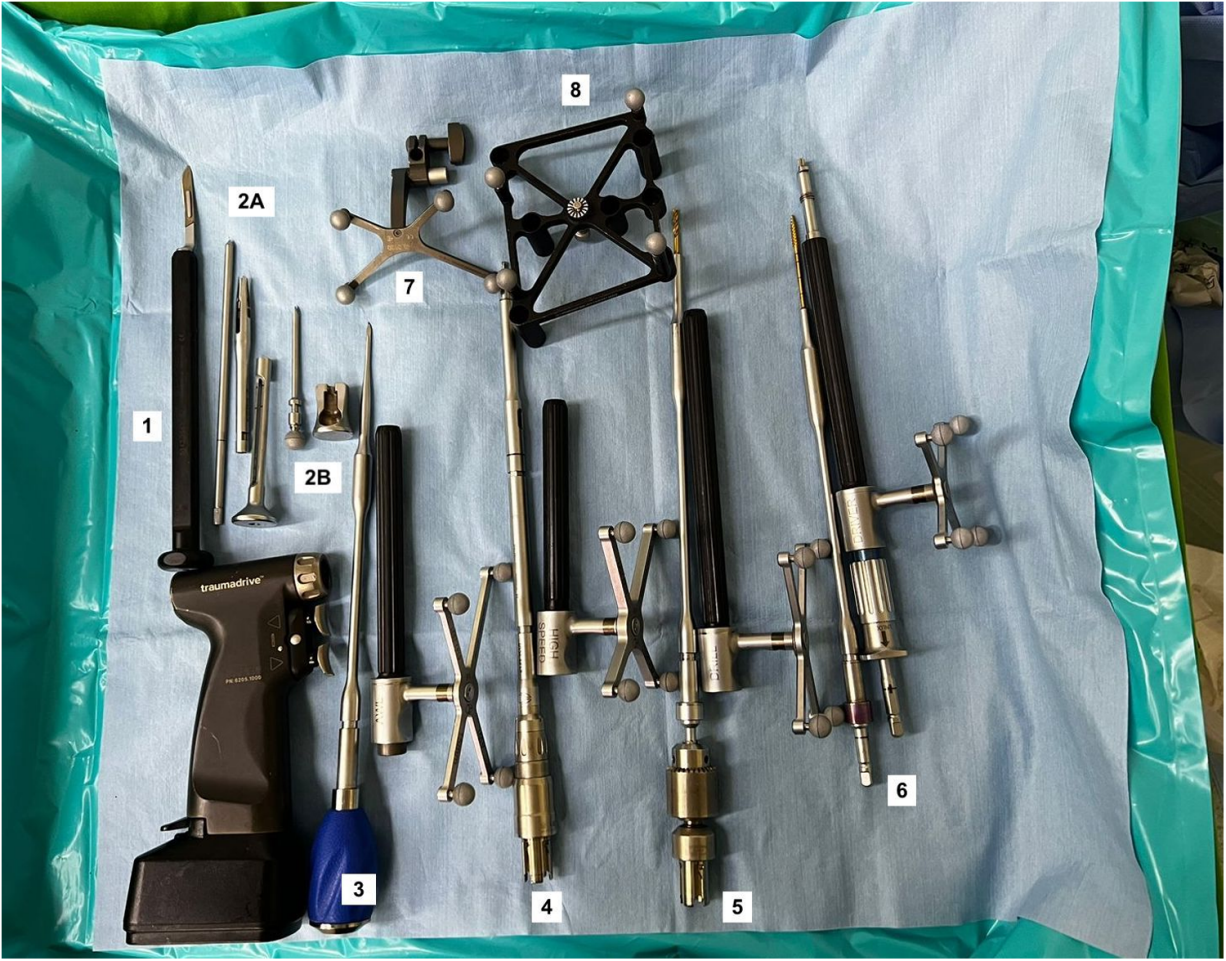
Tools used in the procedure. (1) Scalpel, (2A) Low profile quattro spike, bine clamp, and guide post, (2B) Surveillance marker, (3) Awl and array, (4) Drill and array, (5) High burr and array, (6) Tapper and array, (7) Dynamic Reference Base, (8) Intraoperative Cone-beam Tomography.

In this article, a robotic ExcelsiusGPS (Globus Medical; Audobon, PA, USA) for pedicle screw placement in thoracolumbar surgery was used (Figure 1) (11). The patient is positioned prone on a Jackson table (Figure 2). An area beneath the table is needed to place the image intensifier. Tools used in the procedure are shown in Figure 3.

## Methods

### Surgical planning/workflow

**Figure 4:**
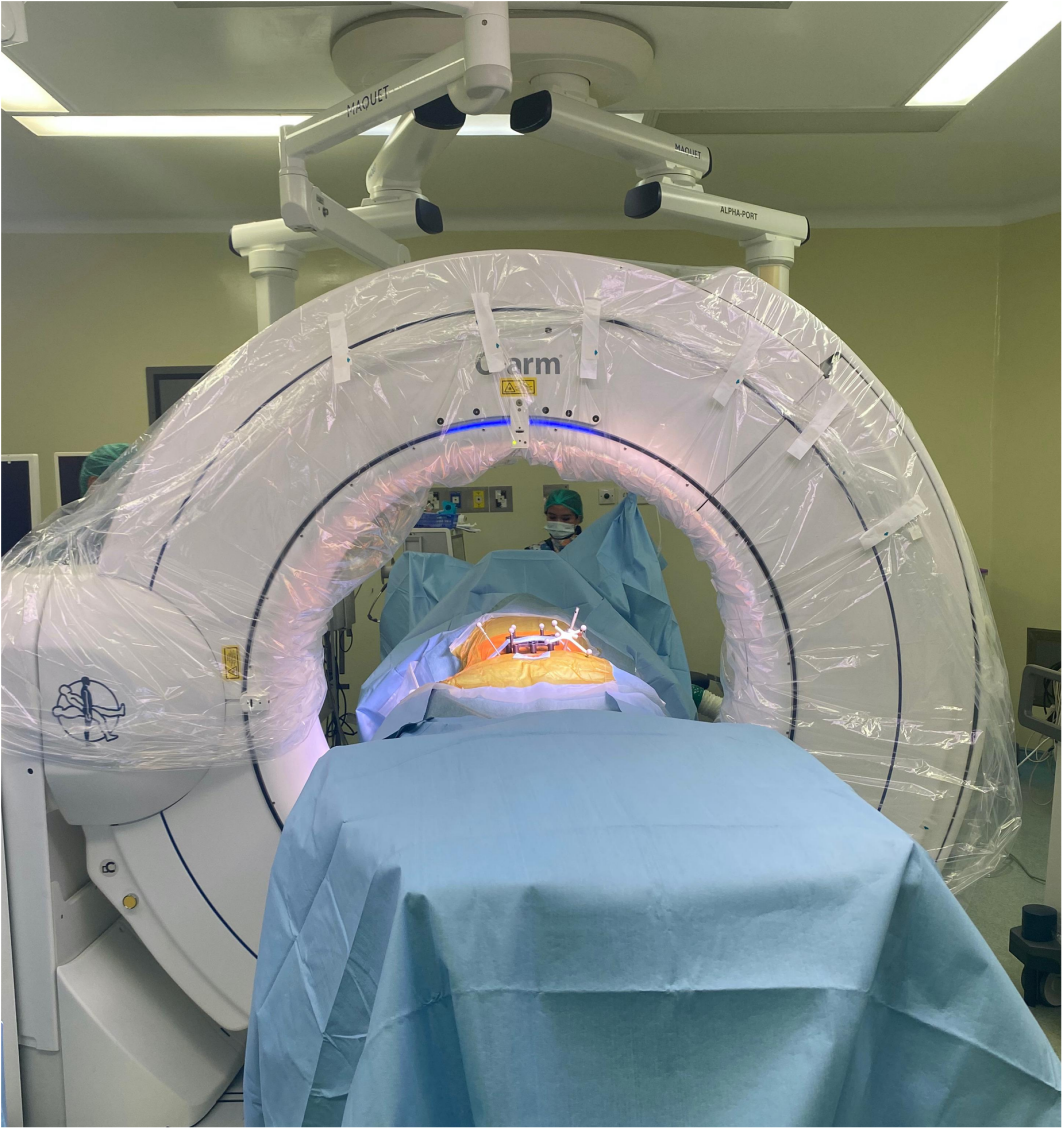
Intraoperative CT scan using O-arm for intraoperative workflow.

Several surgical workflows can be applied: preoperative, fluoroscopy, and intraoperative. This technical note will discuss only the intraoperative workflow. An intraoperative CT scan using O-arm (Medtronic Sofamor Danek, Inc., Memphis, TN) is performed (Figure 4) to allow real-time imaging screw placement planning.

### Placement of dynamic reference base & ICT

**Figure 5:**
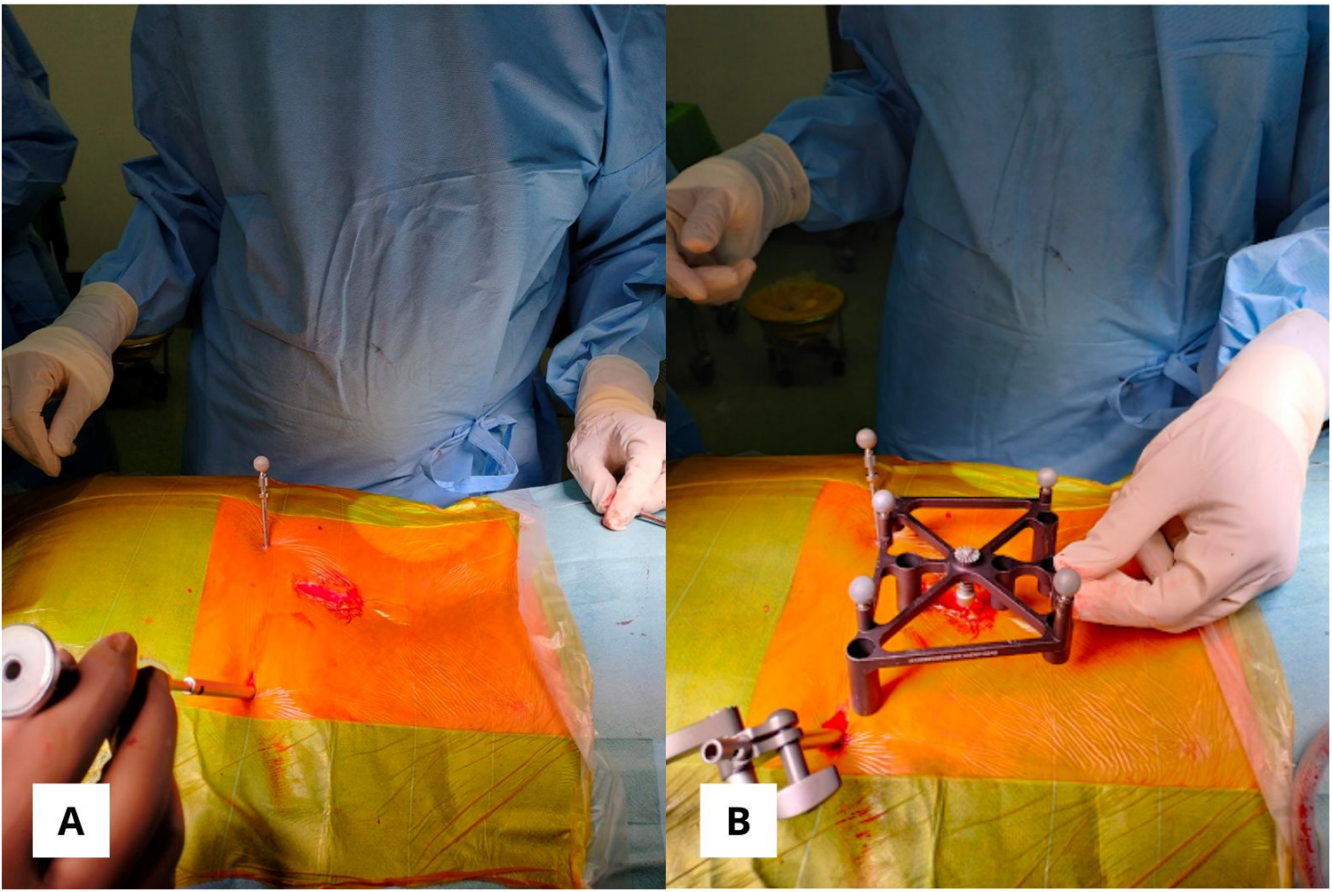
**(A)** The posterior superior iliac spine bilaterally is used for dynamic reference base and surveillance marker positioning. **(B)** ICT (Intraoperative Cone-Beam Tomography) was attached to the spinous process without touching the skin.

The DRB (dynamic reference base) was positioned with a low-profile quattro spike in the right posterior superior iliac spine (PSIS) with a stab incision and placed within 18 cm of the surgical field. The surveillance marker (SM) was placed in the left PSIS (Figure 5). The ICT (Intraoperative Cone-Beam Tomography) was attached to the spinous process and positioned near the desired screw level, parallel to the floor, and as close as possible to the skin without touching it. The DRB and ICT must remain visible to the navigation camera, even after inserting the O-arm. Proper positioning and stability of the DRB, ICT, and surveillance marker (SM), along with verifying anatomical landmarks before screw planning, are crucial for ensuring accuracy and effectiveness during surgery and achieving reliable surgical outcomes.

### Screw planning

**Figure 6:**
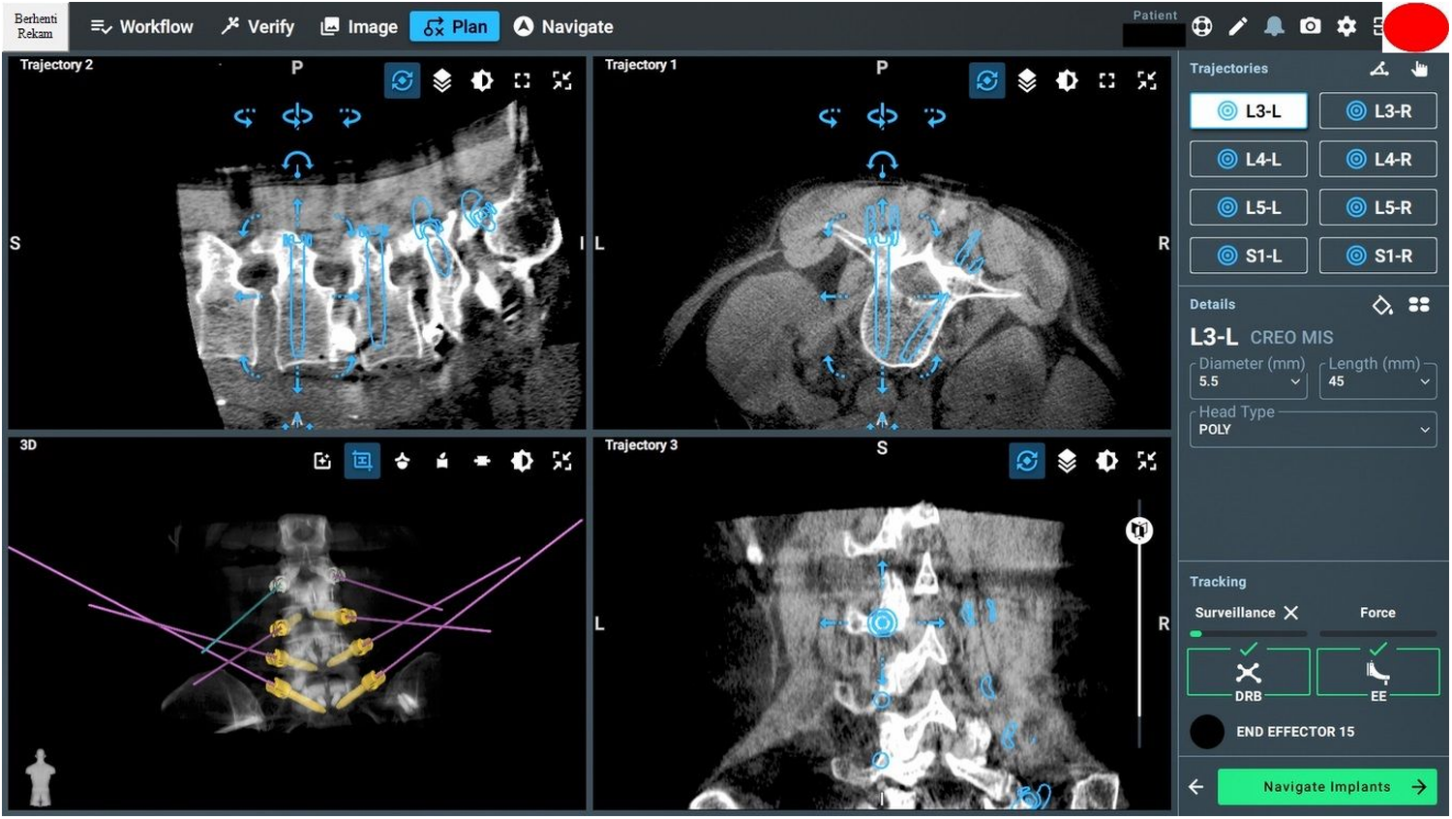
Screw planning includes entry points, trajectories, screw length, and width.

**Figure 7:**
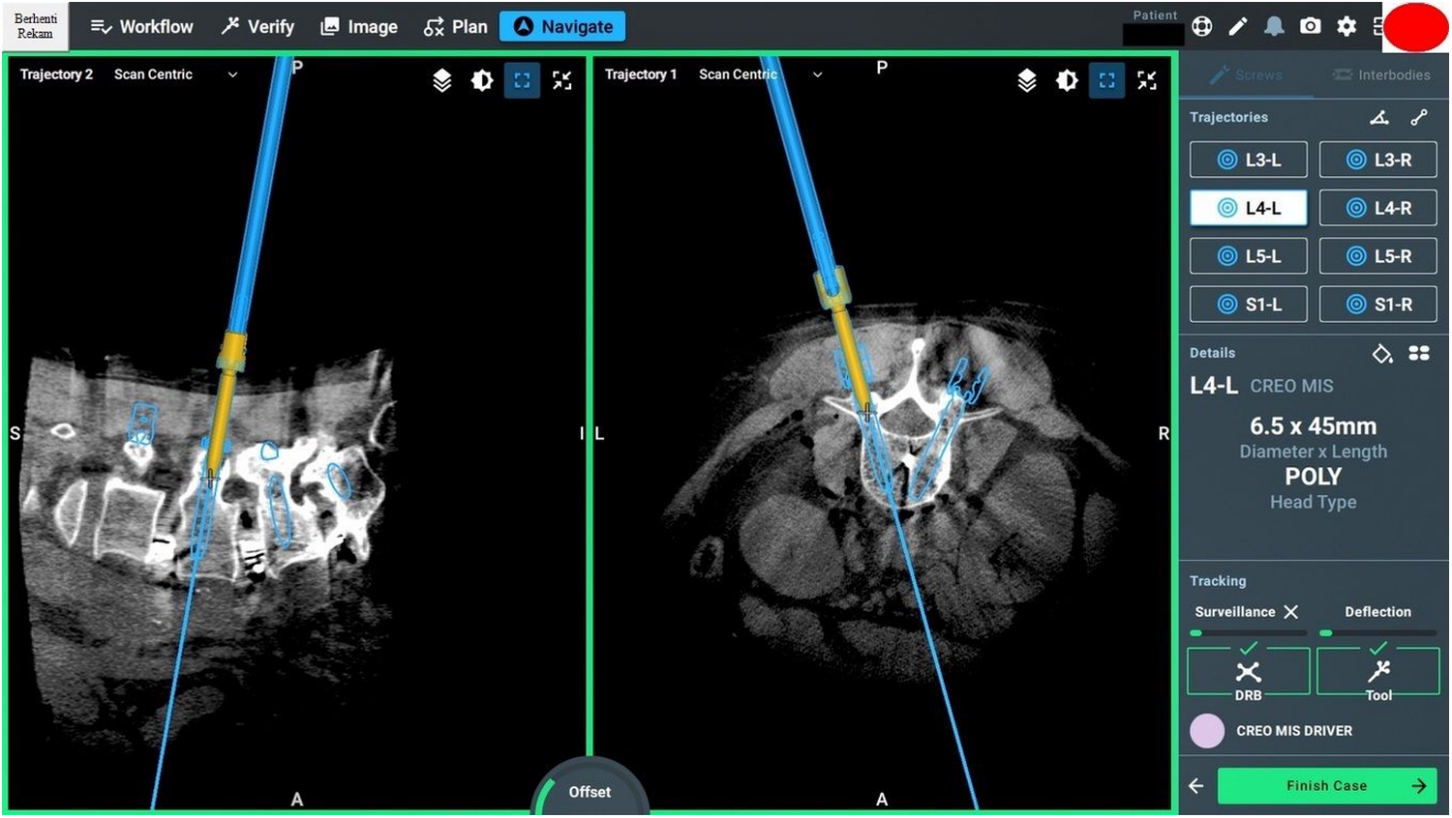
Intraoperative pedicle screw placement using the ExcelsiusGPS robotics system demonstrates the placement of a left-sided L4 pedicle screw.

Effective screw planning using the O-arm and ExcelsiusGPS surgical system involves careful registration of navigated instruments and precise screw trajectory planning to avoid complications (Figures 6, 7). For thoracic screws, it is crucial to prevent the transverse process slope, while lumbar screw planning must account for the superior articular process. Ensuring screw trajectory alignment without collisions and using 3D projections for accuracy are vital steps. In extremely obese patients, adjusting the end effector closer to the skin can help address the challenges posed by increased skin-to-bone distance, ensuring the robotic tools can reach the target effectively.

### Exposure

Once the end effector is in place, the surgeon will mark the skin incision using a scalpel inserted through the guide tube. The end effector is adjusted to accommodate a larger incision. During the incision, ensure that the fascia is adequately dissected beforehand. After the incision, the end effector is repositioned to its original position. The key to ensuring a good trajectory is achieving adequate exposure. Medial excision of the fascia is necessary to enable the proper trajectory for reaching the screw entry point.

### Screw insertion and placement

The initial bur is positioned on the bone at the screw entry point to make an initial hole. The power burr is used before reaching the bone to prevent changes in bone alignment. Screws like Creo MIS screws or K-wire system are placed through the guide tube in the end effector. If using the K-wire system, insert the inner cannula into the guide tube after tapping. Once the K-wire is through the inner cannula, detach the cannula and reposition the end effector. Screws can be inserted with K-wire guidance. The screw is inserted until resistance from the tulip head against the bone is felt.

## Discussion

**Figure 8:**
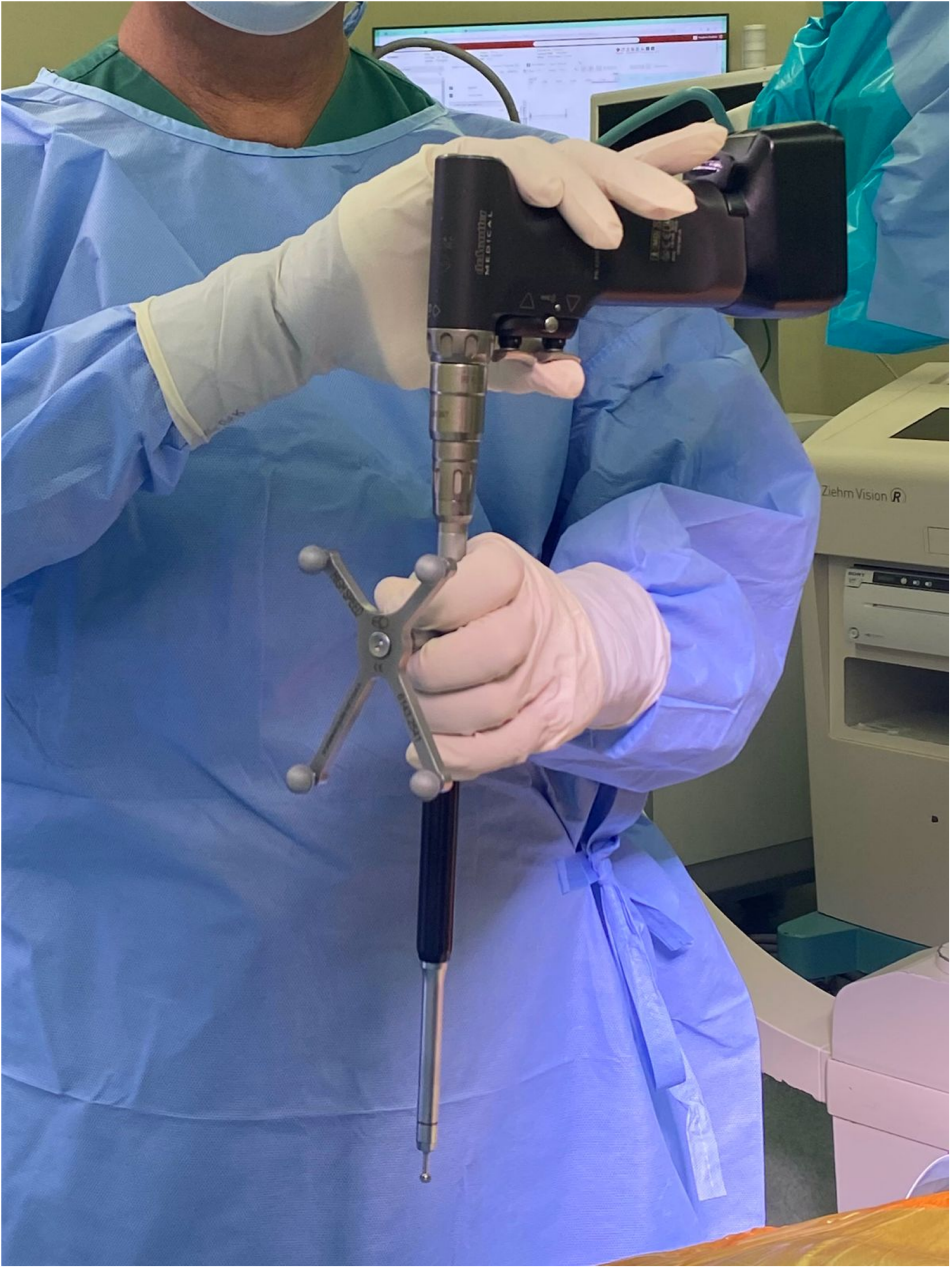
The hamburger grip technique was used to facilitate screw insertion with a power drill system.

Robotic-assisted spine surgery is gaining popularity due to its multiple advantages. However, there are concerns regarding appropriate screw placement under various conditions. In highly flexible bones, alignment often changes with breathing. If this occurs, the anesthesiologist should hold the patient's breath in the expiratory phase, similar to during scanning. During this phase, the initial burr and high-speed drill can be performed. During tapping, the patient can resume normal breathing. After the tapping phase, screw placement follows. Furthermore, during screw placement, tactile feedback and deflection monitoring are crucial for making real-time adjustments and maintaining the instrument's trajectory. Deflection often occurs due to inadequate soft tissue or fascia release and the weight of the power drill. Using a hamburger grip can help counteract this weight (Figure 8).

**Figure 9:**
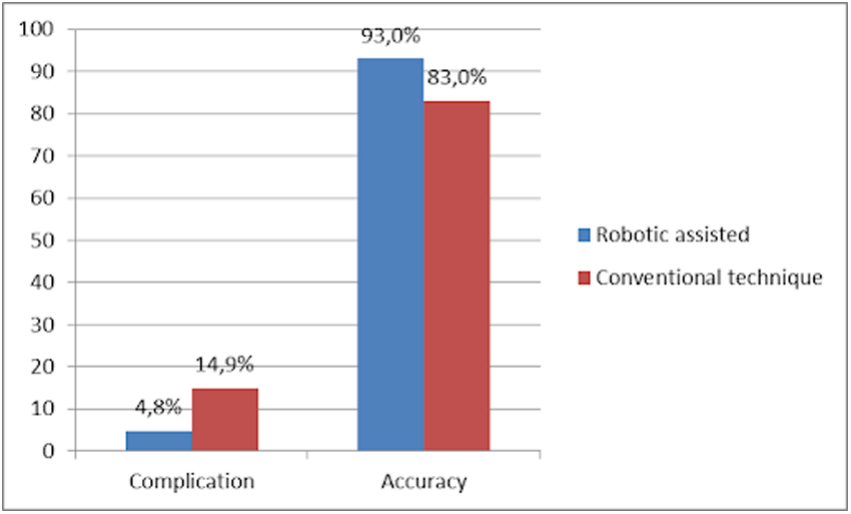
The comparison of outcomes between robotic-assisted and conventional techniques in complication and accuracy (10, 17).

With the growing and widespread adoption of navigation technology, numerous studies have reported superior outcomes and lower complication rates compared to free-hand techniques. A meta-analysis reported fewer complication rates were found in robotic spine-assisted surgery, including less intraoperative blood loss, lower revision rate, fewer complications, and shorter length of hospital stay (12). An extensive meta-analysis of twenty RCTs reported a lower complication rate in the robot-assisted group than in free-hand techniques (4.83% vs. 14.97%) (Figure 9) (10).

Orthopedic surgeons are concerned about radiation exposure. Robotic systems reduce this exposure by avoiding the initial CT scan and minimizing intraoperative radiation. Research shows robotic procedures lower surgeon exposure significantly, with up to a tenfold decrease compared to fluoroscopy-guided techniques (13). Robotic-assisted surgery reduces radiation exposure by 54%–74% compared to conventional CT scans (14).

The precision of pedicle screw placement is crucial, as misplaced screws can lead to devastating vascular, pulmonary, and neurologic complications and even dural leaks (15). A study found that in spine surgery with robot assistance, 91.7% of pedicle screws were accurately placed, with only 6.8% deviating by less than 2 mm (16). Furthermore, most surgeons agree that a screw deviation of ≤2 mm is considered acceptable (15). Yue et al. showed that robot-assisted screw placement is more accurate than fluoroscopy-assisted placement, with a 93% vs. 83% accuracy rate and fewer superior facet joint devastations (Figure 9) (17).

Robotic spine surgery conserves energy for deformity correction, thus enabling less stressful surgery. The utilization of robotic navigation in spine surgery offers a range of advantages and disadvantages (Table 1).

**Table 1:** Advantages and disadvantages of robotic spine surgery.

Advantages	Disadvantages
• Accurate trajectory & size (18)	• Learning curve (15, 19)
• Better implant purchasing (10, 12)	• Slightly longer duration (20)
• Safety (21)	• Increased cost (22, 23)
• Minimize complication (24)	• Tent to put more screws (23)
• Early recovery (24, 25)	
• Lower radiation for the surgeon and the patient (4, 14, 25)	
• Less stressful (10)	
• Conserve energy for deformity correction (24)	
• lower surgeon burn-out (10)	
• Minimally invasive (26)	

### Future directions

The integration of robotic systems in spine surgery is progressing rapidly, with advancements aimed at enhancing efficiency and surgical workflow through improved imaging software and automation. The scope of robotic systems is expected to broaden to include cervical and pelvic fixation and more complex spinal procedures.

## Conclusion

Robotic-assisted spine surgery has improved the accuracy of pedicle screw insertion by providing real-time planning, precise trajectory guidance, and intraoperative adjustments. This enhanced precision reduces complications, including misplacement, while also minimizing radiation exposure to the surgeon and the patient. However, these benefits come with increased costs and a higher usage of screws, necessitating careful evaluation of the procedure's cost-effectiveness and its overall impact on patient outcomes and healthcare resources.

## Data Availability

The raw data supporting the conclusions of this article will be made available by the authors, without undue reservation.
